# Does the Cave Environment Reduce Functional Diversity?

**DOI:** 10.1371/journal.pone.0151958

**Published:** 2016-03-22

**Authors:** Camile Sorbo Fernandes, Marco Antonio Batalha, Maria Elina Bichuette

**Affiliations:** 1 Department of Ecology and Evolutionary Biology, Federal University of São Carlos, PO Box 676, 13565–905, São Carlos, Brazil; 2 Department of Botany, Federal University of São Carlos, PO Box 676, 13565–905, São Carlos, Brazil; University of Cincinnati, UNITED STATES

## Abstract

Caves are not colonised by all taxa present in the surface species pool, due to absence of light and the tendency to food limitation when compared to surface communities. Under strong species sorting during colonisation and later by the restrictive environmental filter, traits that are not adaptive in subterranean habitats may be filtered out. We tested whether cave communities were assembled by the restrictive regime propitiated by permanent darkness or by competitive exclusion due to resource scarcity. When compared to surface communities, the restrictive subterranean regime would lead to lower functional diversity and phenotypic clustering inside the caves, and the opposite should be expected in the case of competitive exclusion. Using isopods (Oniscidea) as model taxa, we measured several niche descriptors of taxa from surface and cave habitats, used a multivariate measure of functional diversity, and compared their widths. We found phenotypic overdispersion and higher functional diversity in cave taxa when compared to surface taxa. On the one hand, the dry climate outside of caves hampered the survival of several taxa and their ecological strategies, not viable under severe desiccation risk, culminating in the clustering of functional traits. In contrast, this restriction does not occur inside of caves, where isopods find favourable conditions under lower predation pressures and more amenable environmental parameters that allow occupation and subsequent diversification. Our results showed that, at least for some taxa, caves may not be such a harsh environment as previously thought. The high functional diversity we found inside caves adds an additional reason for the conservation of these sensitive environments.

## Introduction

All cave communities throughout the world have a remarkable feature in common: the complete darkness in deeper zones, which leads to the absence of photosynthetic producers and to the dependence on surface food intake [1]. As a consequence, there is a tendency toward food shortages and a compromise of all biological processes related to luminosity, such as circadian activity, secretion of hormones, and visual guidance [1]. Due to constraints imposed by these environmental conditions, cave communities are not merely a subset of the surface species pool, but have notable differences when compared to surface communities [2]. For instance, food webs in caves are truncated at the bottom, due to the absence of photosynthetic producers, and at the top, due to the scarcity of specialised predators [3].

Nocturnal and generalist organisms in surface habitats are among the species most likely to colonise caves, as they often can naturally extend their distribution to the subterranean environment [2]. They can either establish source populations in both outside and inside the caves, with individuals moving from one habitat to the other, or give rise to different species, due to vicariance events, with source populations occurring inside the caves [4]. The former are called troglophiles whereas the latter are called troglobionts [4]. Species from the same clade may occur in both habitats unless the surface species becomes extinct, albeit geographic and phylogenetic relicts frequently occur [5]. Preadapted surface taxa may find new challenges to mate and survive inside caves. These environmental stresses combined with founder effects may promote rapid genetic divergence, culminating in parapatric speciation [6, 7].

On the one hand, under strong species sorting during colonisation and, later, strong evolutionary pressures in a restrictive environment, nonadaptive traits may be filtered out in subterranean habitats [8]. The traits most often filtered out are those that are inefficient and costly with respect to fitness in conditions of permanent darkness—diurnal activity, predominant visual orientation, photoautotrophism, and some metabolic paths dependent of light (e.g., calcium absorption in most vertebrates) [1, 2]. In addition, some foraging strategies are not as effective inside caves as they are in surface habitats. In arachnids, for example, sensing web weavers and ambush hunters are not viable, either because the type of web is impossible to build in a cave (sensing web weavers), or the hunting strategy would not be efficient in an habitat with low density of prey (ambush hunters). At the same time, webs useful for crawling insects are favoured in detriment of webs exclusively targeting flying insects, resulting in functional clustering of arachnids inside caves [9]. If so, functional clustering inside the caves may be expected, with morphological, physiological, and behavioral convergences, mainly related to the permanent darkness [1, 10].

Functional overdispersion via niche differentiation, however, has been found in aquatic cave habitats and interstitial groundwater communities [11, 12]. Assembly rules of cave communities are, thus, still unclear. Does the complete darkness act as an environmental filter [8]? Is there competitive exclusion because of resource limitation? [13–15], or are functional traits influenced by random events related to biogeographic legacy [15]?

A means to provide some insights into these questions is by estimating functional diversity, which is based on trait differences among co-occurring species [16]. The more different the traits, the higher the value of functional diversity, because it is based on the sum of the branch lengths of a functional dendrogram [16]. Communities that are functional diverse tend to exhibit greater complementary resource use, and, thus, are expected to operate more efficiently [16]. If environmental filtering is the main ecological force, phenotypic clustering and low functional diversity are expected; if competitive exclusion is the main ecological force, phenotypic overdispersion and high functional diversity are expected [17]. How these traits are organised in a community has antagonistic implications: the higher the functional diversity, the greater the efficiency in resource use, but the lower the resilience of ecological processes [18].

Oniscidean isopods are the most successful crustacean colonisers of land habitats, being a diverse and successful taxon throughout the world, not only in surface but also in subterranean habitats. As such, they are an appropriate model taxon for ecological research in cave biology [19]. In Brazil, for instance, they are abundant and widespread in both surface and subterranean environments [20]. Moreover, most of the documented families include troglomorphic species [20], that is, species bearing apomorphies related to isolation in subterranean environments, as reduced to no eyes, depigmentation, and elongation of appendages [21]. Another advantage presented by the group is that their morphology is strongly associated with ecological strategies of predation and desiccation avoidance [22], interesting characters to be examined in cave communities and in the environmental gradients presented from surface to cave.

Understanding how cave communities are assembled has always been a central problem in speleobiology [11, 12]. Using oniscidean isopods as a model taxon, we aimed to test whether cave communities were assembled by the restrictive regime caused by permanent darkness, leading to phenotypic clustering and lower functional diversity when compared to surface communities, or by limiting similarity caused by competitive exclusion due to resource scarcity, leading to phenotypic overdispersion and higher functional diversity when compared to surface communities. Based on [9], we expected that the restrictive conditions inside the caves would lead to lower values of functional diversity.

## Materials and Methods

### Ethics Statement

This study was approved by the Federal University of São Carlos through the acceptance of a Ph.D. project proposal. The Field Permit was granted by Chico Mendes Institute for Biodiversity Conservation (ICMBIO, 20165–1).

### Study Area

**Fig 1:**
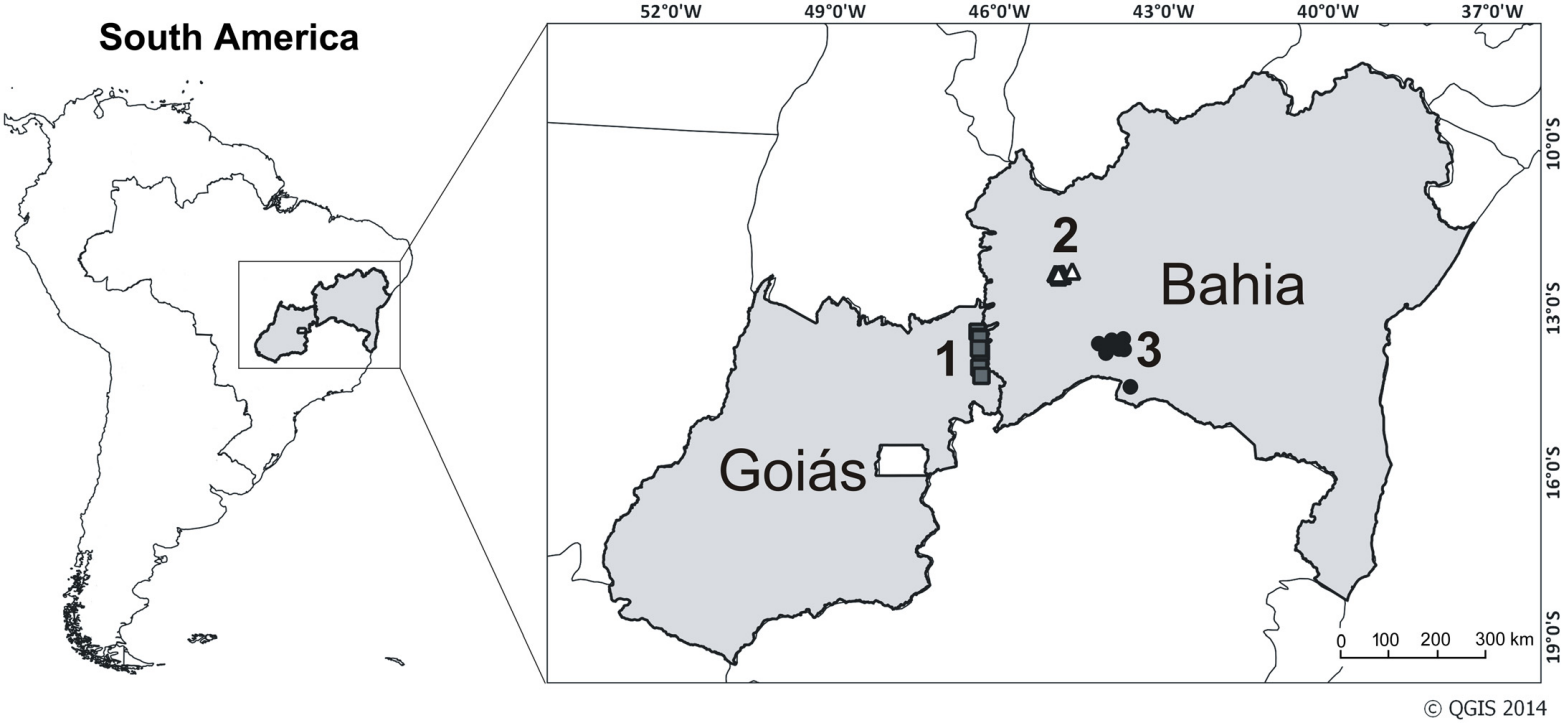
Location of Sampling Sites in Goiás and Bahia States, Brazil. 1. São Domingos, 2. São Desidério, 3. Serra do Ramalho.

We sampled caves distributed along limestone outcrops of similar ages from the Bambuí karstic area, the largest set of limestone outcrops in Brazil [23, 24], located in the Brazilian Central Plateau (Fig 1). We surveyed three subregions in this region, encompassing great complexity of interconnected landscapes. All three subregions occur at similar latitudes and under tropical climate, with wet summers and dry winters (Köppen's Aw) [25]. Two of the subregions, São Desidério and Serra do Ramalho, comprising large extensions of intensively karstified limestone outcrops [23], are located in northeastern Brazil, between the Cerrado and the Caatinga domains [26]. São Desidério comprises at least 200 caves, with large subterranean stream reaches [27]. Large cave systems are also distributed along Serra do Ramalho, which receives abundant input of organic matter through large sinkholes and supports a diverse fauna [28]. The third subregion, São Domingos, is characterized by extensive limestone outcrops, 250 km long and 10–20 km wide [23], and is located in central-western Brazil, in the Cerrado domain [26]. Five large cave systems are known in São Domingos, bearing extensive subterranean drainage and well-developed epikarst, which provide great input of organic matter during the wet season [29]. Together, these subregions comprise a continuum with great biospeleological potential, with many endemic and phylogenetically isolated taxa [20, 29].

### Field Sampling

We collected isopods from 2008 to 2014 in 30 caves and their surrounding surface habitats, distributed throughout the three subregions: 14 in São Desidério, eight in Serra do Ramalho, and eight in São Domingos. Since some caves were located in inaccessible terrain or were prone to flooding, we selected the caves to be sampled based on accessibility and safety. Inside (subterranean) and outside (surface) each cave, with a 4-hr sampling effort (2-hr each habitat type), we took opportunistic samples of several types of microhabitats, including aquatic microhabitats, by active search, that is, turning over rocks, logs, and debris. Once the search was completed, we restored all rocks, logs, and debris to their original location, avoiding unnecessary impacts. Resource inputs inside the caves consisted mainly of vegetal debris brought from the surface by rainfalls during wet season, forming several spots of humid, decomposing leaves, branches, and twigs explored by isopods. Bat guano was of secondary importance as food source in most cave systems.

The farther from the entrance, the less influence of the surface on the cave, because of the absence of light and buffering effect caused by the rocks. Three main cave zones are defined based on this gradual reduction of surface influence: the light zone—near the entrance, with direct influence of light, high temperature variations, and where surface and cave fauna tend to occur together; the twilight zone—with indirect incidence of light and less influence of outside than the entrance; and the aphotic zone, permanently in complete darkness, with gradual transition to stable temperature and air humidity near the saturation [30]. With exception of the entrance (light zone), we checked all accessible habitat zones of each cave using equal amount of time in each zone [30].

Outside the caves, we removed at least 5 cm of soil at several locations within around 100 m of the cave entrance, searching for surface species, and sampled leaf litter, using Winkler extractors and Berlese funnels [31]. We took approximately ten soil samples, and collected leaf litter using a shovel or hands, near of the locations of soil samples. We sampled around three bags with 20,000 cm^3^ of leaf litter each per cave and then, processed it in the traps while still in the field base.

The relatively shallow depth of soil removal results from the dry and compacted soil from the study area. Even though soil-dwelling isopods can migrate vertically throughout the day, they usually are not very good diggers, and, consequently, do not have an important role in soil structure. This superficial distribution of isopods is particularly true in shallow tropical soils [22]. That is the reason most ecological studies with isopods access only the litter and the superficial soil layer (e.g.,[32]).

Among the collected specimens, 101 individuals were from São Desidério, 64 from Serra do Ramalho, and 94 from São Domingos. We also received 76 individuals from Serra do Ramalho as a donation from the University of São Paulo. We accessioned all specimens in the Federal University of São Carlos, where they were morphotyped based on morphology and scanning electron microscopy. Some specimens were later sent to specialists for identification and description of the new species. For simplicity, we refer to these operational taxonomic units as OTUs throughout the text.

Selection and measurement of functional traits

**Fig 2:**
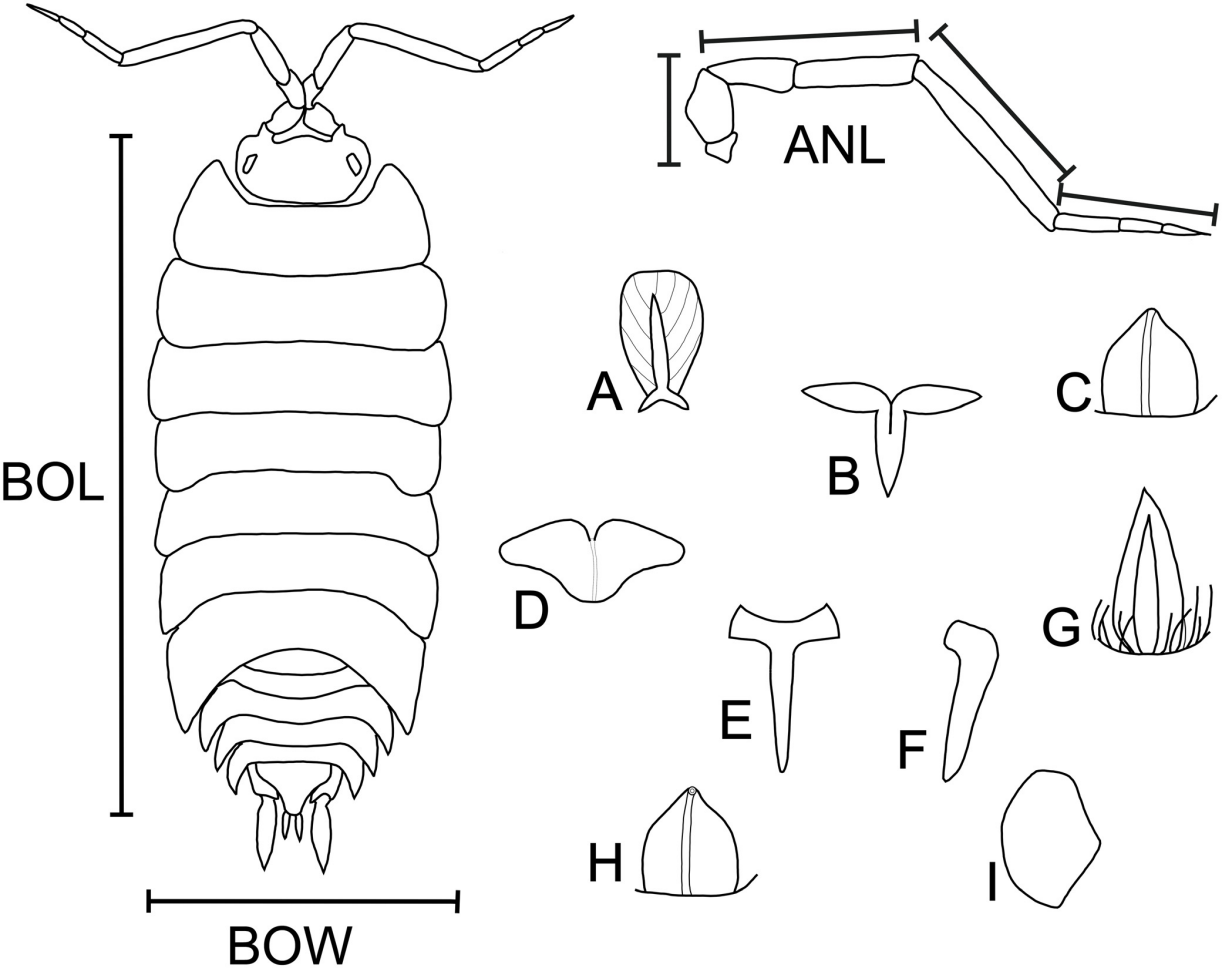
Body Variables and Types of Mechanoreceptors. BOL = body length, BOW = body width, ANL = antenna length, A = fan-shaped scale seta, B-D = tricorn, E-F = tricorn-like, G = lanceolate, H = foraminate tricorn, I = squat tricorn.

For each OTU, we measured the following functional traits (Fig 2): (1) body length, (2) body width, (3) antenna length, (4) volvation (based on morphology), (5) pigmentation (freshly collected specimens), (6) number of ommatidia, (7) number of mechanoreceptors (mean density per mm^2^), (8) type of mechanoreceptors (based on[33]), (9) habit (based on [22]), and (10) type of substratum. These traits are related to habitat exploitation, such as the number and type of mechanoreceptors, body size, and antenna size [33, 34]; predator avoidance, such as habit and volvation [22]; foraging, such as antennae length and type of substratum [35]; spatial orientation, such as antenna length, number of ommatidia, and number and type of mechanoreceptors [36]; or reduction of stabilising selection due to absence of light, such as pigmentation and number of ommatidia [30, 37]. By definition, functional traits are directly or indirectly related to resource use. In the latter case, resource use complementarity can be measured by assuming that traits relate to resource capture differences among species [16].

We dehydrated the collected specimens in an ethanol series, with gradually higher concentrations (80%, 96%, 100%) until critical point and then estimated the number and type of mechanoreceptors using low vacuum scanning electron microscopy [38], grouping them according to their shape [36]. Using a stereo microscope, we also examined volvation, pigmentation, and habit [22, 33, 36]. We assigned substratum type based on field observations.

### Data analysis

Independently of being troglophile or troglobite, all species living inside the caves contribute with resource use and community processes. When using the multivariate index of functional diversity proposed by [16], the definition of guilds can be made *a posteriori*, using UPGMA. This process reduces the subjectivity of guild placement. Consequently, the functional diversity of all OTUs inside the caves was compared to the functional diversity of all surface OTUs.

We constructed a community matrix, with OTUs in rows, environment (surface or subterranean) in columns, and presence or absence in cells. We also constructed a trait matrix, with OTUs in rows, traits in columns, and average values of traits in cells. When an OTU occurred in both environments, we calculated two averages, one for surface individuals and another for subterranean individuals. Based on the trait matrix, we constructed a distance matrix between OTUs, using Gower distances [39]. We clustered the distance matrix, using unweighted pair group method with arithmetic averages, to produce the functional dendrogram. We calculated functional diversity as the total branch length in the dendrogram connecting all OTUs in either the surface or the subterranean environment [16]. We compared the observed difference in functional diversity of the two environments with a pseudo-distribution of 999 values, generated by finding the difference between two random environments with the same number of OTUs as the observed surface and subterranean ones. For this comparison, we calculated the deviation of the observed value from the mean of the null distribution and then divided the values by the standard deviation of the null distribution (standardized effect size). The score is where the observed value lands in the null distribution and can be used to estimate the significance values [40].

Using the trait matrix, we also performed a principal component analysis (PCA) [41] and constructed an ordination diagram, distinguishing surface and subterranean OTUs. As we used scaling 2, we could also establish the correlation between traits [42]. We then selected which principal components represented interpretable variations, using broken-stick model plots [42]. We conducted all analyses in R [43].

## Results

**Fig 3:**
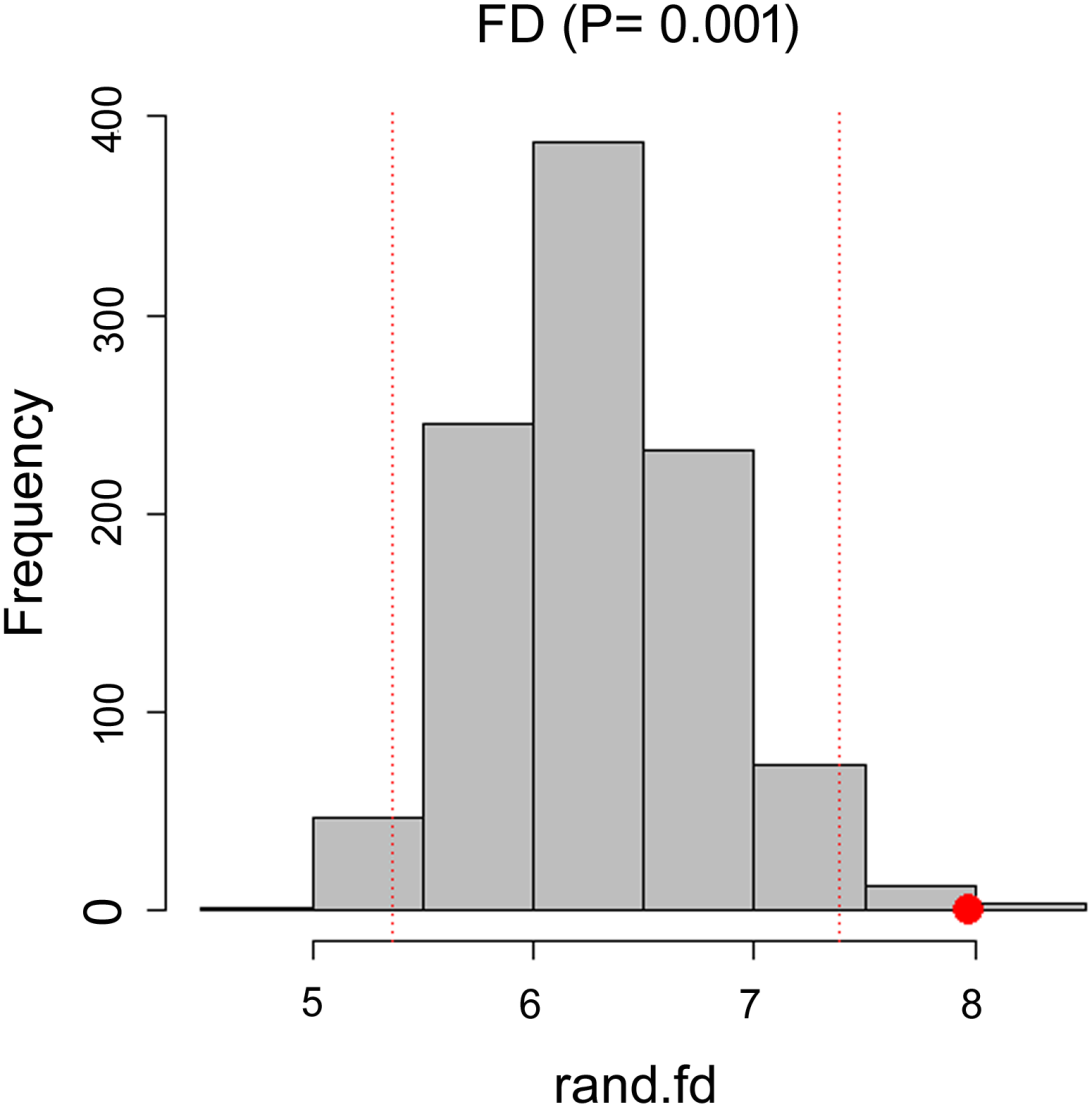
Value of Significance for α = 5%. The red dot shows where the observed value lands in the null distribution of random communities.

We analysed 335 individuals, belonging to 27 OTUs, for which we measured functional traits (Table 1). Three OTUs were found exclusively outside the caves, 20 exclusively inside, and four occurred in both surface and cave habitats. Functional diversity outside the caves was lower than inside (FD_subterranean_ = 11.42; FD_surface_ = 3.45) and the difference between them was significant (P < 0.001) (Fig 3).

**Table 1:** Sampled OTUs, Number of Individuals, and Values of Functional Traits.

OTU	Env	N	BOL	BOW	ANL	VOL	PIG	OMT	NME	TME	HAB	SUB
Armadillidae sp. 1	H	1	3.64	1.30	1.09	1	0.5	12	800	6	4	7
Armadillidae sp. 2	E	2	5.55 (±0.064)	1.87 (±0.20)	1.54 (±0.052)	1	1	16	600	2	4	7
Dubioniscidae sp. 1	H	31	3.28 (±0.62)	1.18 (±0.23)	1.32 (±0.23)	0	1	10	800	1	2	11
Dubioniscidae sp. 1	E	5	3.24 (±0.34)	1.23 (±0.14)	1.29 (±0.16)	0	1	10	800	1	2	7
Dubioniscidae sp. 2	H	10	3.89 (±0.14)	1.55 (±0.31)	1.72 (±0.38)	0	1	12	600	1	2	6
Dubioniscidae sp. 3	H	10	3.28 (±1.32)	1.35 (±0.59)	2.02 (±0.94)	0	0.5	13	500	1	1	6
Dubioniscidae sp. 4	E	1	1.88	0.87	0.82	0	1	8	1200	1	3	7
Dubioniscidae sp. 5	H	1	2.10	0.69	0.93	0	0	0	800	3	2	7
Dubioniscidae sp. 6	H	8	4.02 (±1.09)	1.51 (±0.37)	2.02 (±0.43)	0	0.5	12	800	1	1	11
Dubioniscidae sp. 7	H	12	3.67 (±1.49)	1.44 (±0.59)	1.97 (±0.84)	0	1	14	500	1	2	11
Dubioniscidae sp. 7	E	1	4.72	1.7	2.47	0	1	14	500	1	2	7
Dubioniscidae sp. 8	H	4	3.59 (±1.038)	1.30 (±0.43)	1.80 (±0.69)	0	1	10	500	1	1	7
Dubioniscidae sp. 9	E	1	3.45	1.39	1.45	0	1	13	400	3	2	6
Dubioniscidae sp. 10	H	5	3.68 (±0.51)	1.37 (±0.21)	2.20 (±0.22)	0	1	15	400	1	2	7
*Microsphaeroniscus* sp. 1	H	73	2.61 (±0.57)	0.92 (±0.20)	0.64 (±0.15)	1	0	0	600	2	4	10
*Pectenoniscus* sp. 1	H	8	2.36 (±0.52)	0.86 (±0.22)	0.71 (±0.14)	0	0	0	200	5	6	3
Scleropactidae sp. 1	H	1	3.37	0.99	0.80	1	0.5	0	500	2	4	10
Scleropactidae sp. 2	H	1	16.18	5.4	3.35	1	1	12	200	2	4	2
Styloniscidae sp. 1	H	2	2.93 (±0.71)	0.89 (±0.25)	0.9 (±0.30)	0	0	0	1600	3	2	5
*Trichorhina* sp. 1	H	16	2.51 (±0.64)	0.85 (±0.21)	0.84 (±0.23)	0	0	0	400	1	3	11
*Trichorhina* sp. 2	H	24	2.48 (±0.88)	1.02 (±0.37)	0.80 (±0.29)	0	0	0	1000	1	3	1
*Trichorhina* sp. 3	H	36	2.42 (±0.4)	1.05 (±0.20)	0.90 (±0.13)	0	0.5	2	400	1	1	3
*Trichorhina* sp. 4	H	7	2.71 (±0.72)	1.13 (±0.24)	0.97 (±0.12)	0	0	4	500	1	1	7
*Trichorhina* sp. 5	H	1	1.43	0.44	0.57	0	0	0	3000	1	2	6
*Venezillo* sp. 1	H	10	8.84 (±2.15)	3.88 (±0.99)	2.67 (±0.61)	1	1	20	500	2	4	1
*Venezillo* sp. 1	E	5	7.07 (±0.97)	2.79 (±0.32)	1.78 (±0.22)	1	1	20	500	2	4	11
*Venezillo* sp. 2	H	29	6.06 (±2.0)	2.23 (±0.77)	1.69 (±0.49)	1	1	15	700	2	4	11
*Venezillo* sp. 2	E	3	6.87 (±1.39)	2.62 (±0.51)	1.86 (±0.40)	1	1	15	700	2	4	7
*Xangoniscus aganju*	H	1	7.37	2.93	2.51	0	0	0	200	4	5	9
*Xangoniscus* sp. 1	H	7	8.55 (±1.25)	3.32 (±0.51)	3.26 (±0.53)	0	0	0	150	1	5	9
*Xangoniscus* sp. 2	H	18	8.13 (±1.17)	3.46 (±0.56)	2.87 (±0.58)	0	0	0	200	1	5	9

Env = Environment, E = surface (epigean), H = subterranean (hypogean); N = number of examined specimens; BOL = body length (±standard deviation); BOW = body width (±standard deviation); ANL = antenna length (±standard deviation); OMT = number of ommatidia; VOL = volvation, 0 = absent, 1 = present; PIG = pigmentation, 0 = depigmented, 0.5 = light, 1 = normal; HAB = habit, 1 = clingers, 2 = runners, 3 = non-conformists, 4 = rollers, 5 = clinger-rollers, 6 = amphibious, 7 = creepers; TME = type of mechanoreceptors, 1 = scale seta, 2 = tricorn, 3 = tricorn-like, 4 = lanceolate, foraminated tricorn, 6 = flat tricorn; NME = number of mechanoreceptors; SUB = type of substratum, 1 = dry clay under sliver of limestone, 2 = wet clay and roots, 3 = frugivorous bat guano, 4 = carnivorous bat guano, 5 = rock and gravel and leaf litter, 6 = rock, 7 = leaf litter, 8 = rock and consolidated sediment, 9 = submerged organic matter, 10 = sediment banks with millipedes feces and vegetal debris, 11 = several, that is, if a OTU was found in a variety of substrate types.

**Fig 4:**
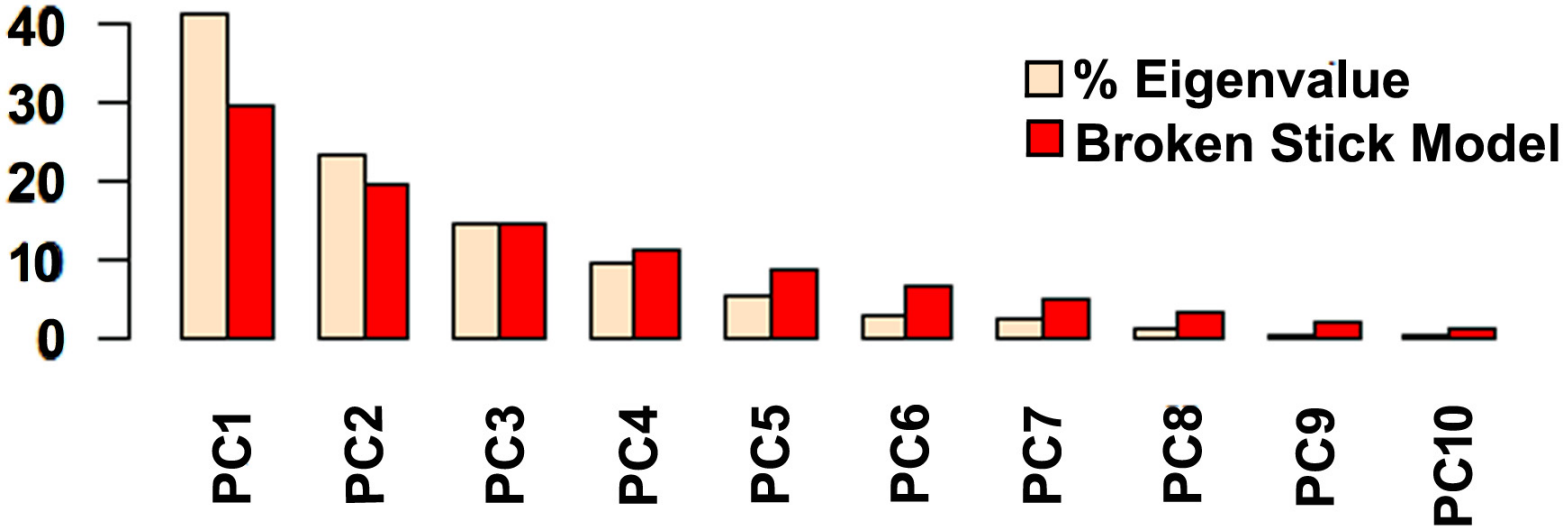
Broken-Stick Model Plots to Help Assess the Number of Interpretable Axes in PCA.

**Fig 5:**
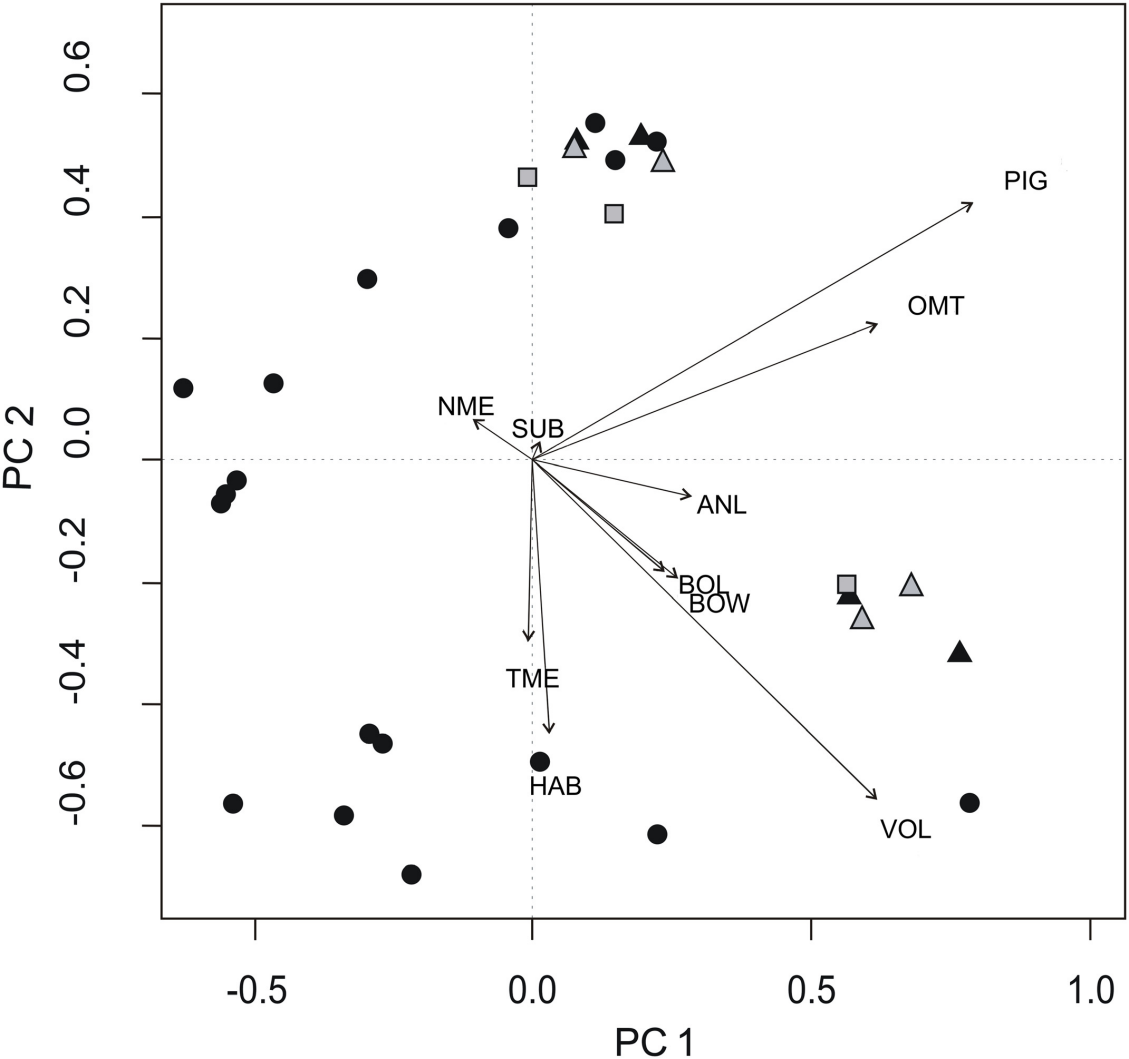
Ordination Diagram of Surface (Grey) and Subterranean (Black) OTUs, According to their Functional Traits. Triangles represent taxa occurring in both environments, surface ones in grey and subterranean ones in black. BOL = body length, BOW = body width, ANL = antenna length, VOL = volvation, PIG = pigmentation, OMT = number of ommatidia, NME = number of mechanoreceptors, TME = type of mechanoreceptors, HAB = habit, and SUB = type of substratum.

According to the Broken Stick Model applied to the eigenvalues (Fig 4), axes 1 and 2 represented interpretable structures, with 64.19% of variation explained. The other eight did not differ from random variance. Surface OTUs formed two groups, one related to large body size and volvation and another related to the presence of ommatidia and pigmentation, whereas subterranean OTUs were scattered (Fig 5).

The first principal component (PC1) was positively linked to traits related to progressively dryer environments. At the lower right corner of the biplot (Fig 5), there were larger and volvation taxa, all of them troglophiles or found exclusively outside the caves. At the opposite side of PC1, there were exclusively subterranean taxa, with the greatest variation of mechanoreceptor types and habits (PC2) (Fig 5).

## Discussion

Our sampling highlighted the Linnean and Wallacean shortfalls for Brazilian isopods, respectively the lack of formal taxonomic descriptions and gaps in our understanding of geographical distributions [44]. Taxonomic impediment is a problem for many cave studies, because new undescribed species are frequently found and taxonomists are scarce for several taxonomic groups, elevating the time spent in descriptions and identification [45]. In such cases, the use of operational taxonomic units, as we employed in this study, is justifiable [45]. Species identification in our study was limited because of the time spent in measuring functional traits before they could be sent and dissected by a taxonomist. OTUs have been sent to specialists for proper identification and description. Our ecological conclusions remain valid, because functional diversity is independent of species identity [16].

We found greater functional diversity inside caves than outside in surface habitats, suggesting phenotypic overdispersion (Fig 5). Since our null model took into account the number of OTUs in each environment, greater functional diversity inside caves was not caused or influenced by greater species (OTU) diversity.

Volvation, as seen in the lower right corner of the PC1 (Fig 5), in surface or troglophile taxa, is a strategy strongly related to avoiding desiccation and predators [22, 46]. The higher values of types of mechanoreceptors and habits on the opposite side of the PC1 corresponds, respectively, to lanceolate and foraminated mechanoreceptors and to amphibious or creeper habits, related to advantageous strategies in high humidity environments [22, 33]. Subterranean taxa also had the highest numbers of mechanoreceptors, related to orientation in the dark [33] and the lowest values of ommatidia and pigmentation. These traits are correlated and, with a few exceptions related to endogeous lifestyle, indicate cave specialization [47]. It is interesting to note that all ecological strategies except volvation were found predominantly inside the caves (Fig 5). Even isopods being found in various substratum types, it had little importance in explaining the distribution of surface and subterranean taxa in trait space, probably because organic matter inside the caves consisted almost exclusively in a humid subset of surface resources.

In surface habitats, temperatures are high, the dry season is long, and humidity is low; conditions that are important environmental constraints for terrestrial crustaceans [48]. Thus, climate could act as an environmental filter, restricting the number of species able to colonise surface habitats, decreasing the number of viable strategies, and, ultimately, decreasing functional diversity. Conditions inside caves are more stable, with lower temperatures and higher humidity, more favourable for terrestrial crustaceans. These milder conditions, coupled with the absence of specialised predators [3], may contribute to increased functional diversity inside caves.

Cave organisms are capable of coping with darkness and the relative unpredictability of resource input, since they belong to lineages that possess traits which offer advantages in the colonisation of subterranean habitats, the so-called “preadaptations” or “exaptations” [2]. Among the most important traits distinguishing subterranean from surface species are troglomorphisms, such as the reduction or absence of pigmentation and ommatidia, changes in body dimensions, and higher density of mechanoreceptors [21, 47]. Desiccation risk and predation are negligible pressures in subterranean habitats, which could explain the predominance of volvation forms outside the caves. Except for the caves in Serra do Ramalho, volvation ability was present in OTUs that occurred either outside the caves or in both environments, but no troglobiont presented this ability.

Decreased functional diversity inside than outside of caves is more likely in predators or lineages that originate in drier environments. For instance, lower values of functional diversity inside the caves than outside were found for spiders in the Iberian Peninsula of Europe [9]. In caves, predators are limited by the dependence of the invertebrates filtered from the species pool, leading to more restricted diets and the elimination of some predation strategies [3, 9]. In the Iberian Peninsula, however, only five traits were examined, four of them categorical, which could have influenced the quality of the functional space and led to a biased assessment of functional diversity [49]. We examined ten traits, half of them were continuous, which are expected to provide a good representation of the functional space [49]. Moreover, as detritivores, isopods are not limited by prey disponibility. The high number of subterranean taxa, many with narrow geographical ranges, suggests that caves harbor favorable conditions that promote colonization and subsequent diversification in Brazilian isopods. Isopods with very similar niches may be competitively excluded from a particular cave. It is not uncommon to find multiple species with wide distributions not co-occurring in the same cave, and never have we observed two species exploiting the same resource in the same cave. On occasion, there is no trait convergence as the environment becomes harsher [34], which may be attributed to fine-level niche partitioning [12]. Our results showed that this pattern should be more generally accepted, and some putative environmental filters, such resource limitation and darkness, may be compensated by other favourable environmental conditions. At least for some taxonomic groups, caves may not be such a harsh environment as previously thought.

Environmental filters can obfuscate the phylogenetic history if the traits are convergent in different clades. Consequently, the relationship between phylogenetic diversity and functional diversity will depend on how conserved the traits are [15, 17]. This is an interesting avenue for future studies in Brazilian isopods, but the species diversity and phylogenetic relationships of this group must first be elucidated.

Resource use by isopods inside caves tends to be more efficient than in surface habitat, leading to higher values of functional diversity. This higher complementarity implies that there is a fine niche adjustment and higher vulnerability of environmental processes if species composition changes [18, 50]. Based on our results, as well as in the great faunistic relevance already discussed in previous studies [20], the study areas should be priority sites for conservation. Even if São Domingos is inside a conservation unit, the integrity of subterranean environments is still vulnerable because the headwaters of all subterranean streams and rivers are unprotected and is threatened by sedimentation and pollution [51]. Both Serra do Ramalho and São Desidério do not have any form of legal protection and have been facing threats mainly related to agricultural and urban expansion [20, 52, 53]. Current Brazilian laws only effectively preserve caves classified as maximum level of relevance. Amongst the biological criteria, the maximum relevance is only reached when a cave is habitat for rare or troglobitic species (decree 6640/2008 and its normative instruction), which are inherently fragile and should be at least in the category “vulnerable” of IUCN (International Union for Conservation of Nature) [51]. Conservation acts, though, require the description of the species and their inclusion in lists of endangered species, demanding genomic, population, and systematic studies to understand the category the species should be included. For these reasons, taxonomic studies of subterranean fauna in Brazil have crucial importance for the preservation of both the subterranean environment and biodiversity [28]. We are currently making efforts to create Conservation Units for São Desidério and Serra do Ramalho, in view of the great biological relevance coupled with growing anthropic pressure these areas have been facing. Several rare, troglobitic and endemic species already were described for these areas [20] and others will be included at the time of their description, emphasizing the national relevance of these caves. The high functional diversity of isopods we found inside the caves adds an additional reason for the conservation of such fragile environments.
